# Traumatic brain injury—needs and treatment options in the chronic phase: Study protocol for a randomized controlled community-based intervention

**DOI:** 10.1186/s13063-020-4195-5

**Published:** 2020-03-27

**Authors:** Ida Maria H. Borgen, Marianne Løvstad, Nada Andelic, Solveig Hauger, Solrun Sigurdardottir, Helene L. Søberg, Unni Sveen, Marit V. Forslund, Ingerid Kleffelgård, Marte Ørud Lindstad, Laraine Winter, Cecilie Røe

**Affiliations:** 1 Department of Physical Medicine and Rehabilitation, Oslo University Hospital, Oslo, Norway; 2 Department of Psychology, Faculty of Social Sciences, University of Oslo, Oslo, Norway; 3 Department of Research, Sunnaas Rehabilitation Hospital, Nesoddtangen, Norway; 4 Center for Habilitation and Rehabilitation Models and Services (CHARM), Institute of Health and Society, University of Oslo, Oslo, Norway; 5 Faculty of Health Sciences, Oslo Metropolitan University, Oslo, Norway; 6 Department of Health Sciences in Gjøvik, Faculty of Medicine and Health Sciences, Norwegian University of Science and Technology, Gjøvik, Norway; 7 Philadelphia Research and Education Foundation, Philadelphia, PA USA; 8 Nursing Service, Department of Veterans Affairs Medical Center, Philadelphia, PA USA; 9 Institute of Clinical Medicine, Faculty of Medicine, University of Oslo, Oslo, Norway

**Keywords:** Brain injury, In-home rehabilitation, Community-based rehabilitation, Chronic phase, Health-care services, Outcome measures

## Abstract

**Background:**

Traumatic brain injury (TBI) is often associated with life-long medical, cognitive, emotional, and behavioral changes. Although long-lasting disabilities are expected, research on effective treatment options in the chronic phase of TBI is scarce.

**Methods/design:**

This study protocol describes a randomized controlled trial (RCT) aimed at evaluating the effectiveness of a goal-oriented and community-based intervention for increasing community integration, quality of life, and functional independence in the chronic phase of complicated mild to severe TBI. Participants will be recruited from Oslo University Hospital, Norway. Patients aged 18–72 years living at home with MRI/CT-verified intracranial abnormalities, a TBI diagnosis, a time since injury of ≥ 2 years, and who experience either current TBI-related problems or restrictions in community integration will be included. The 120 participants will be randomized 1:1 to either (a) an intervention group, which will receive an in-home intervention program over 4 months, or (b) a control group receiving standard care in the municipalities. The intervention will consist of six home visits and two telephone contacts with a rehabilitation professional. A SMART-goal approach will be adopted to target the individual’s self-reported TBI difficulties in everyday life. Primary outcomes will be self-reported quality of life and participation. Secondary outcomes include symptom burden, emotional functioning, and clinician-assessed global outcome and need for rehabilitation services. Outcomes will be evaluated at baseline and 4–5 and 12 months after baseline. Caregiver burden and general health will be assessed in participating family members. Goal attainment and acceptability will be evaluated in the intervention group. A process evaluation will be carried out to evaluate protocol adherence, and a cost-effectiveness analysis will be applied if the intervention is found to be effective.

**Discussion:**

The current study provides an innovative approach to rehabilitation in the chronic phase of TBI evaluated using an RCT design that may inform treatment planning, health policies, and coordination of patient care. Further, the study may demonstrate new modes of establishing collaboration and knowledge transition between specialized rehabilitation facilities and local rehabilitation services that may improve patient outcomes.

**Trial registration:**

ClinicalTrials.gov, NCT03545594. Registered on June 4th, 2018.

## Administrative information

Note: the numbers in curly brackets in this protocol refer to SPIRIT checklist item numbers. The order of the items has been modified to group similar items (see http://www.equator-network.org/reporting-guidelines/spirit-2013-statement-defining-standard-protocol-items-for-clinical-trials/).

**Table:** 

Title {1}	Traumatic brain injury: needs and treatment options in the chronic phase. Study protocol for a randomized controlled community-based intervention.
Trial registration {2a and 2b}.	ClinicalTrials.gov, NCT03545594. Registered on June 4^th^, 2018. https://clinicaltrials.gov/ct2/show/NCT03545594
Protocol version {3}	07/02/2020, version 3.0.
Funding {4}	The project is funded by the Research Council of Norway, project number 260673/H10.
Author details {5a}	^1^Department of Physical Medicine and Rehabilitation, Oslo University Hospital, Norway. ^2^Department of Psychology, Faculty of Social Sciences, University of Oslo, Norway. ^3^Institute of Clinical Medicine, Faculty of Medicine, University of Oslo, Norway. ^4^Department of Research, Sunnaas Rehabilitation Hospital, Nesoddtangen, Norway. ^5^Center for Habilitation and Rehabilitation Models and Services (CHARM), Institute of Health and Society, University of Oslo, Norway. ^6^ Faculty of Health Sciences, Oslo Metropolitan University. ^7^Department of Health Sciences in Gjøvik, Faculty of Medicine and Health Sciences, Norwegian University of Science and Technology. ^8^Philadelphia Research and Education Foundation. ^9^Nursing Service, Department of Veterans Affairs Medical Center, Philadelphia, PA, USA
Name and contact information for the trial sponsor {5b}	Not applicable.
Role of sponsor {5c}	Not applicable.

## Background

### Rationale {6a}

Traumatic brain injury (TBI) is associated with life-long medical, cognitive, emotional, and behavioral changes and is a leading cause of death and disability worldwide [1, 2]. An estimated 3.17 million people in the United States alone are living with TBI-related disabilities [3], and estimates for the European Union are approximately 7.7 million individuals [4, 5]. Research has demonstrated persistent difficulties in areas including cognitive, vocational, and emotional functioning, as well as reduced quality of life and community integration at both 3–5 [6–10] and 10 years’ post-injury [11–14]. Some experts have argued that TBI should be thought of as a chronic disease process, indicating that a long-term perspective is necessary when planning and providing health-care services for individuals with TBI [15, 16].

Although a large knowledge base exists regarding treatment in the acute and sub-acute phases of TBI [17–20], we are still in the early stages of bringing rehabilitation programs closer to community services and in providing the needed rehabilitation in the chronic phase. Reports from user organizations point towards a major dilemma in TBI treatment, in that extensive medical treatment is provided only in the early phases, after which many patients feel that they are left to deal with chronic adversity on their own [21]. A Norwegian study showed that 5 years after moderate to severe TBI, approximately one-third of the individuals reported their self-perceived health-care needs were unmet [22]. Further, services offered in the chronic phase most often target physical functioning, whereas needs related to cognitive, emotional, and vocational difficulties are more often unmet [9, 23–26]. Despite these trends in service delivery, several studies have documented the efficacy of rehabilitation programs aimed at remediation of specific domains, such as memory, attention, and executive and emotional functioning [19, 27].

Following TBI, there is a need to consider the patient’s functioning and goals with an ecological perspective in the community, as impaired functional competency and restrictions in participation are more visible in the patient’s living and social environments than in clinical settings. The patient’s self-defined problems and goals of care should be targets of intervention. These individual preferences, in addition to environmental support from the family and social networks, must be aligned in order to improve treatment relevance, motivation, and adherence [28]. Furthermore, the living environment should be a target for intervention to match the patient’s level of competency if needed [29]. The role of the home environment in everyday function and well-being is based on Lewin’s person–environment fit concept [30], which concerns the interaction between personal competence and environmental press (i.e., the demands from the environment that support or challenge performance of daily activities). A good fit between the person’s competence and environmental press results in optimal outcomes—positive affect and adaptive behavior. When an individual’s competence is impaired (as with chronic TBI), the range of acceptable environmental press becomes narrower. Because environment forces may either support or create a barrier to positive outcomes, the home environment should be targeted for intervention. Despite this, health-care and social-support services are rarely individually tailored or delivered in the patient’s home environment, and high quality controlled studies targeting the effects of community-based rehabilitation are scarce [31, 32]. Further, although rehabilitation services in the acute and sub-acute phase are often delivered in a specialized rehabilitation setting, rehabilitation services in the chronic phase are typically delivered by primary health-care professionals. The World Health Organization’s 2030 rehabilitation strategy [33] encourages a strong cooperation between different levels of health care to ensure effective and more integrated rehabilitation services for users. Systematic knowledge transition from specialized rehabilitation services to the primary-care services is considered essential to ensure coherency in rehabilitation services provided in different phases of TBI.

Hence, the current study aims to evaluate an in-home rehabilitation program tailored to the individual’s TBI-related difficulties in the chronic phase. This randomized controlled trial (RCT) was inspired by a home-based rehabilitation study by Winter et al. that included 81 veterans with TBI in a two-group RCT [29]. While the control group received treatment as usual (TAU), the intervention group followed an eight-session, home-based rehabilitation program delivered in the veterans’ homes and in close collaboration with a family member. The intervention was person-centered, focusing on targeted activity problems identified by the veterans, and used an action plan that included goals and tailored strategies to fit the individual’s physical and social environments. Their study documented the efficacy of the in-home program guided by the person–environmental fit model and showed significantly higher community re-integration and less difficulty managing targeted problems in the treatment group, compared with controls. However, since the study only included military veterans with TBI, the authors emphasized the need for replication with civilians. Almost 70% of participants in Winter et al.’s study had mild TBI, and additional investigation is needed in larger populations, including individuals with moderate-to-severe TBI. Furthermore, the Winter et al. study did not include long-term follow-up or process or cost-effectiveness evaluations. Finally, health-care delivery and social-security systems, as well as culture, differ between countries. For instance, Norway is a welfare state with a public health-care system and may not be comparable to the US veteran system. Hence, the study protocol by Winter et al. was adapted according to cultural issues and differences in the target population. The aim of the current study is to evaluate a community-based, individualized, and goal-oriented intervention targeting civilians with complicated mild to severe TBI in Norway.

### Objectives {7}

Our specific hypotheses are:

- H1: Person-centered intervention targeting the participant’s problems in functioning in their living environment will result in improved quality of life and participation compared with treatment as usual (TAU).
- H2: Person-centered intervention will result in a lower burden of self-reported TBI-related problems compared with TAU.
- H3: Person-centered intervention will result in improved physical and mental health compared with TAU.
- H4: Person-centered intervention will result in fewer unmet health-care needs compared with TAU.
- H5: Person-centered intervention will be a cost-effective alternative compared with TAU.
- H6: Patients, family members, and rehabilitation professionals involved will be satisfied with the intervention program.

### Trial design {8}

**Fig. 1:**
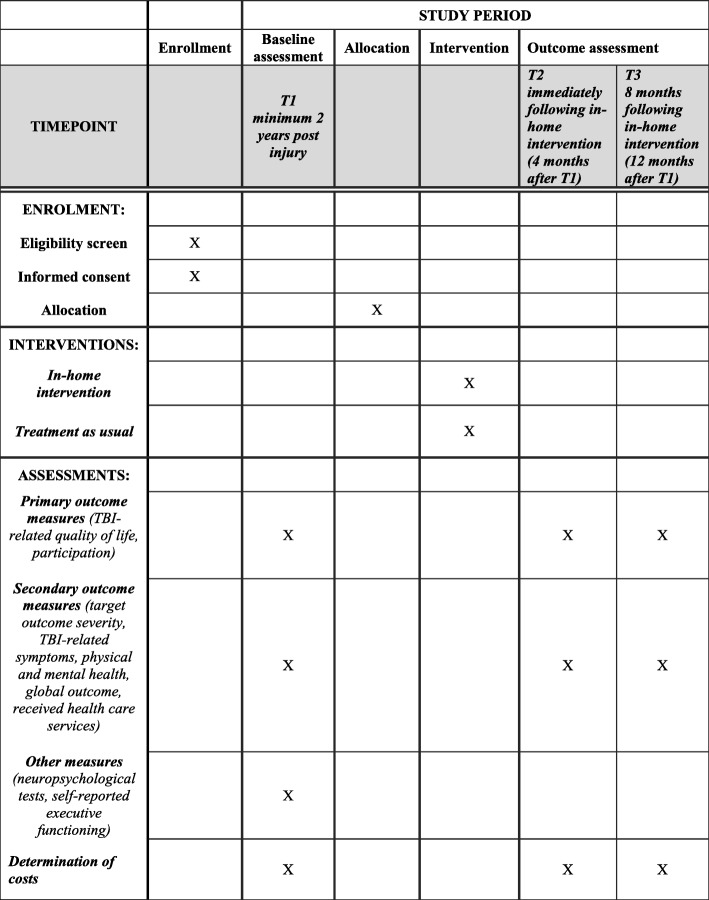
Standard Protocol Items: Recommendations for Interventional Trials (SPIRIT)

The study is a two-group RCT with a mixed-methods design. Figure 1 displays standard protocol items according to the Standard Protocol Items: Recommendations for Interventional Trials (SPIRIT) [34, 35]. Potentially eligible participants will be invited by letter and screened by phone for inclusion and exclusion criteria. A baseline assessment (T1) will be conducted using measures of cognitive, emotional, and physical functioning as well as functional competence, participation, current use of health-care services, and main activity problems. Subsequently, participants will be randomized to intervention or TAU groups. Further assessments will be carried out 4–5 (T2) and 12 months (T3) after baseline. The timing of the T2 assessment will be aimed to correspond to the approximate end of the intervention for the intervention group. Use of health-care services will be registered and mapped according to the International Classification System for Service Organization in Health-related Rehabilitation (ICSO-R) [36] over the study period in both groups.

In line with the new Medical Research Council guidance [37], a feasibility study [38] was conducted to evaluate inclusion criteria, feasibility of intervention manual, and outcome measures, as well as acceptability. The feasibility study included six individuals with severe TBI, and intervention delivery was concluded in June 2018.

## Methods/design

### Study setting {9}

Oslo University hospital (OUH) is the trauma referral center in South-East Norway, serving more than half of the Norwegian population (> 2.5 mil). Assessments will be conducted at an outpatient clinic at OUH, and intervention sessions will be delivered in the participant’s home. The intervention may also be delivered at the outpatient TBI clinic at OUH if requested by the participant.

### Eligibility criteria {10}

The study will include patients from OUH with a TBI diagnosis and CT/MRI-verified intracranial abnormalities. Participants must be 18–72 years of age at inclusion, ≥ 16 years of age at the time of the injury, at least 2 years’ post-injury at study inclusion, and living at home. The participants must report ongoing TBI-related cognitive, emotional, and/or physical problems, and/or reduced physical and mental health, and/or difficulties with participation in activities with family, friends, and/or in the community (based on interview and the standardized questionnaires at baseline). If the participants have a family member or friend closely involved in their lives, the family member/friend will be asked to participate as well. Participants with severe progressive neurologic disorders or severe psychiatric disorders that would confound outcome assessments will be excluded as well as those unable to provide informed consent or participate in a goal-setting process. Participants with insufficient fluency in Norwegian to allow for communication with therapists and outcome assessors or that have active substance abuse or violent tendencies that would put therapists at risk during home visits will also be excluded.

### Patient characteristics

The following sociodemographic variables will be recorded at baseline: age, gender, marital status, living arrangement, educational level, and current employment status. Medical variables will be obtained from the medical journal and include comorbidity, injury characteristics, and clinical severity (Glasgow Coma Scale Score, length of posttraumatic amnesia), neuroimaging results, and primary rehabilitation services received. A neuropsychological test battery will be conducted at baseline (T1) to assess cognitive functioning and guide intervention strategies. The battery consists of tests of abstract reasoning (Similarities and Matrix Reasoning from the Wechsler Adult Intelligence Scale (WAIS-IV) [38]), verbal learning and memory (California Verbal Learning Test-II [39]), and attention span (Digit Span, WAIS-IV [40]) as well as processing speed, mental flexibility, and inhibition (Trail Making Tests and Color Word Interference Tests from the Delis-Kaplan Executive Function System (D-KEFS) [41]). A questionnaire regarding executive functioning in everyday living will also be administered at T1 (the Behavior Rating Inventory of Executive Function Adult Version (BRIEF-A) Self-Report [42]).

If inclusion of a family member is possible, participants will answer a short questionnaire pertaining to the quality of their relationship with their family member (adapted version of the Quality of Relationship scale used by Winter et al. [29]) and the family member will be asked to fill out the BRIEF-A Informant Form [42].

Careful consideration has been given to the selection of neuropsychological tests and questionnaires included for patient characteristics in relation to patient burden, and order of administration will be standardized and checked for missing data during administration.

#### Who will take informed consent? {26a}

Signed written informed consent forms will be collected from all participants and participating family members by the therapist conducting the baseline assessment.

#### Additional consent provisions for collection and use of participant data and biological specimens {26b}

Not applicable.

### Interventions

#### Explanation for the choice of comparators {6b}

A comparison group receiving treatment as usual was chosen to assess whether the intervention is better or at least equivalent to current clinical practice in Norway (see “Background and rationale”).

#### Intervention description {11a}

##### Patient-centered intervention

**Fig. 2:**
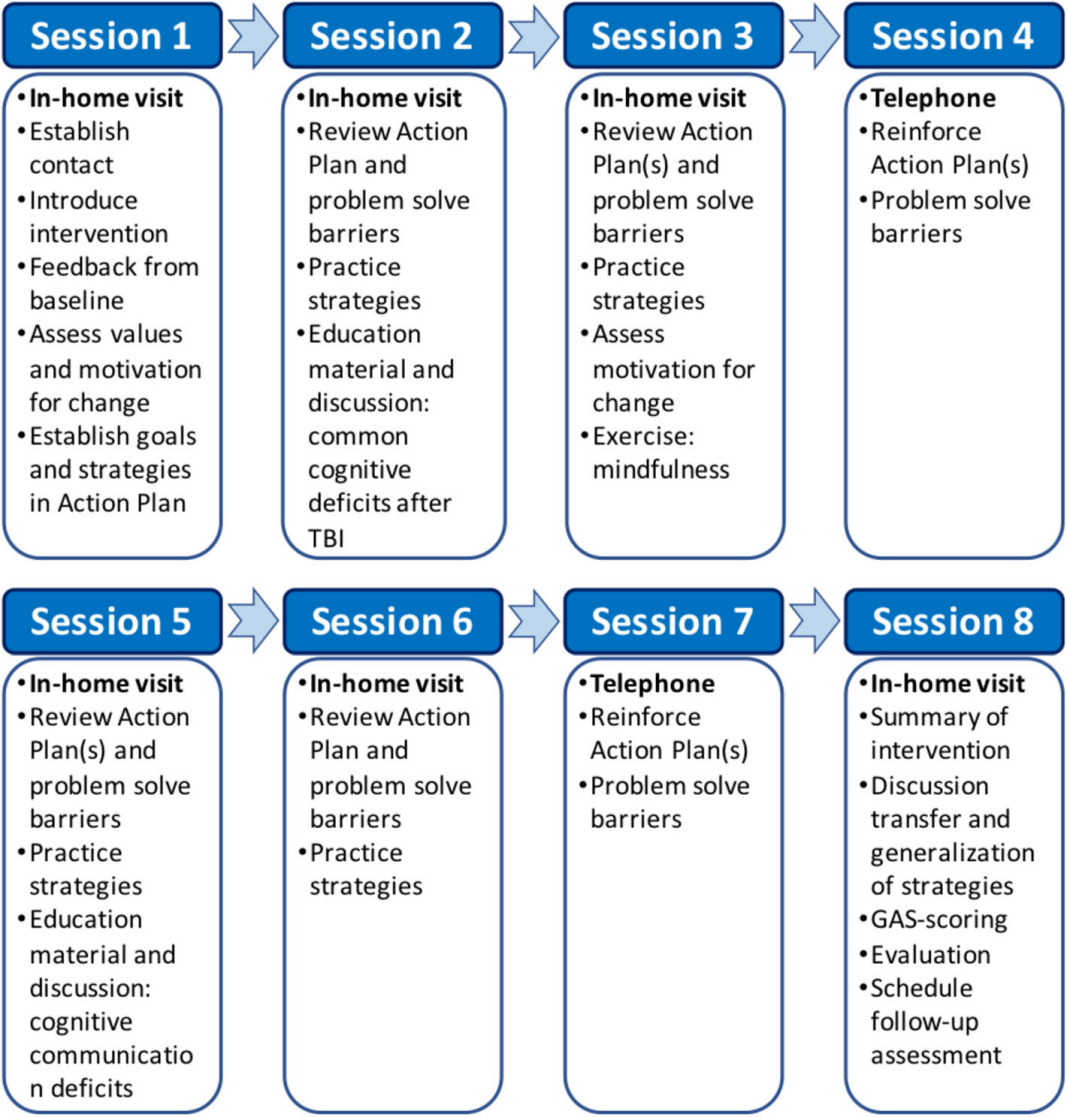
Overview of intervention sessions

The intervention is modeled after the Winter et al. study [29] and will consist of eight sessions (six in-home visits of approximately 2-h duration and two telephone contacts). The intervention will be delivered over a period of approximately 4 months and, when possible, in collaboration with a family member/friend who is involved in the participant’s everyday life. An overview of the intervention sessions is displayed in Fig. 2.

To increase the proficiency of the goal-setting process, a SMART-goal approach will be used. SMART goals need to be specific, measurable, achievable, realistic/relevant, and timed [43]. Goal attainment scaling (GAS) [44] will be applied during the establishment of SMART goals to provide a quantifiable measure of goal achievement at the end of the intervention.

The intervention will be conducted in three phases: (1) identification of target problem areas (target outcomes) that disrupt activities of everyday life; (2) establishment of SMART-goals and GAS; and (3) development of an action plan containing evidence-based strategies to mitigate the reported problems, including environmental modifications and compensatory strategies. The intervention sessions will include strategy training, identification of obstacles to goal achievement, and guidance in generalization and transferability of new skills. The manual provides a framework for the intervention; however, the specific content of the action plan will be highly individualized, as it is based on problem areas nominated by the participant.

Based on the most commonly occurring long-term symptoms after moderate-to-severe TBI [8–14, 23] in addition to the experiences of Winter et al. [29] and our feasibility study, target outcomes and defined SMART goals are expected to be related to the following: cognitive (e.g., memory, attention, executive functioning, self-awareness, and social communication); physical (e.g., sensory and motor deficits, fatigue, dizziness, sleep disorders, reduced balance, and visual problems); emotional (e.g., anxiety or depressive symptoms secondary to injury, and stress management); and interpersonal problems (e.g., reduced awareness of deficits, personality changes, disinhibited behavior, apathy, and irritability). To ensure high-quality interventions, the study will include components from evidence-based treatment programs within relevant functional domains, enabling the adaptation of specialized rehabilitation programs to the home setting. Interventions in the areas of memory, attention, executive functioning, symptom awareness, and social communication will be provided according to recommendations by the Cognitive Rehabilitation Task Force [27] and the INCOG Guidelines for Cognitive Rehabilitation following TBI [45–48], as well as the recommendations by the American Congress of Rehabilitation Medicine [49]. Muscle relaxation and mindfulness techniques will be used to address problems with stress management. Regarding symptoms of anxiety and depression, techniques derived from cognitive behavioral therapy [50] and behavior activation [51] will comprise the main theoretical approaches, although an eclectic stance will be taken (e.g., in cases when threats to identity and self-concept are seen to be more readily addressed using other therapeutic approaches) [52–55]. All participants will be provided with hand-out materials and psychoeducation concerning common cognitive impairments in the chronic phase of TBI, cognitive communication difficulties, and an introduction to mindfulness exercises as a stress management technique.

When relevant and feasible, family members or local health professionals who are involved in the care of the participants will be invited to participate during the intervention sessions. At baseline assessment, participants can nominate their primary local health-care professional to join the intervention sessions if they wish. For participants without a local health-care professional but considered to be in need of establishing contact with primary care services, the therapist will establish such contact in collaboration with the patient’s general practitioner to ensure lasting knowledge transference.

Four therapists (a psychologist, neuropsychologist, physician, and physiotherapist) will be responsible for the delivery of the intervention.

##### Treatment as usual

The control group will continue to receive their usual health-care and rehabilitation services provided in the municipality. In Norway, the municipalities are mainly responsible for follow-up in the chronic phase of TBI. This follow-up will potentially vary greatly depending on the needs of the individual and what municipality they live in, ranging from no follow-up to regular contact with local rehabilitation teams. The services provided for each individual in the control group will be thoroughly logged at all follow-ups to allow comparison with the intervention group regarding content, professionals involved, etc. Any concurrent treatment of this type will not be discontinued in any group due to ethical considerations.

#### Criteria for discontinuing or modifying allocated interventions {11b}

All therapists are trained health-care professionals and rehabilitation professionals. Any cases of adverse effects of the intervention will be discussed in the research group, and suitable actions for the participant in question will be ensured. If signs of severe psychiatric symptoms, including suicidal ideation, are detected during contact with participants, the therapist will immediately consult with senior researchers who are specialist medical doctors and psychologists. Procedures for this are part of the manual.

#### Strategies to improve adherence to interventions {11c}

The principal investigators in collaboration with senior TBI researchers will supervise the therapists. Further, senior researchers will evaluate treatment fidelity by attending 10% of all in-home visits and will attempt to detect and alert to possible bias reflecting therapists’ professional backgrounds. Any need for adjustments in the protocol will be discussed and resolved in project meetings throughout the project period.

#### Relevant concomitant care permitted or prohibited during the trial {11d}

Participants will not be withdrawn from any concurrent treatment during the trial.

### Provisions for post-trial care {30}

Need for further follow-up will be evaluated in the control group after the end of the trial, and they will be referred and treated accordingly.

### Outcomes {12}

The primary outcome measures are measures of participation (PART-O) [56] and TBI-specific quality of life (QOLIBRI) [57]. Secondary outcomes include the severity of target problem areas (target outcomes), goal attainment, need for rehabilitation services, global outcome, symptom burden, physical and mental health, self-awareness, and satisfaction with the intervention. All outcome measures will be administered at all time points (T1–T3), and order of administration will be standardized. Table 1 provides a list of all instruments that will be used as outcome measures, including references to their psychometric properties. To assess goal achievement and satisfaction with the intervention, two measures (acceptability-scale and GAS scores) can only be measured in the intervention group. Although comparison with the control group is not possible on these measures, they will still provide important information regarding goal attainment and treatment acceptability. The selection of outcome measures has been thoroughly planned according to patient and family member acceptability and time needed for completion.

**Table 1:** Outcome measures

Outcome measure	Measures
Primary outcome measures	
Participation	Participation Assessment with Recombined Tools- Objective (PART-O) [56, 58]
Quality of life	Quality of Life After Brain Injury (QOLIBRI) Overall Scale [57, 59]
Secondary outcome measures	
Individually identified target functional domains and their severity	Target outcomes and their severity, as rated on a Likert scale from 0 to 4 (0 = not difficult at all, 4 = extremely difficult), based on Winter et al. [29]
Goal achievement^*^	Goal Attainment Scaling (GAS) [44]
Symptom burden	Rivermead Post-Concussion Questionnaire (RPQ) [60]
Needs for rehabilitation and social support	Needs and Provision Complexity Scale-Clinician version [61, 62]
Global outcome	Glasgow Outcome Scale-Extended (GOSE) [63, 64]
Emotional functioning (depressive and anxiety symptoms)	Patients Health Questionnaire (PHQ-9) [65] Generalized Anxiety Disorder (GAD-7) scale [66]
Physical and mental health and quality-adjusted life years (QALYs)	EQ-5D [67]
Competency in daily activities	Patient Competency Rating Scale (PCRS) Patient Form [68, 69]
Acceptability of intervention assessed by patient, family member and health professional*	Acceptability Scale (Scale used by Winter et al., adapted and translated into Norwegian) [29]
Family member outcomes	
Participant’s competency in daily activities, participant’s self-awareness	PCRS Relative Form [68, 69]
Caregiver burden	Caregiver Burden Scale [70]
Family member depressive symptoms	PHQ-9 [65]
Family member general health	EQ-5D VAS-scale (0 = worst health possible, 100 = best health possible) [67]

* Only assessed in the intervention group

### Participant timeline {13}

**Fig. 3:**
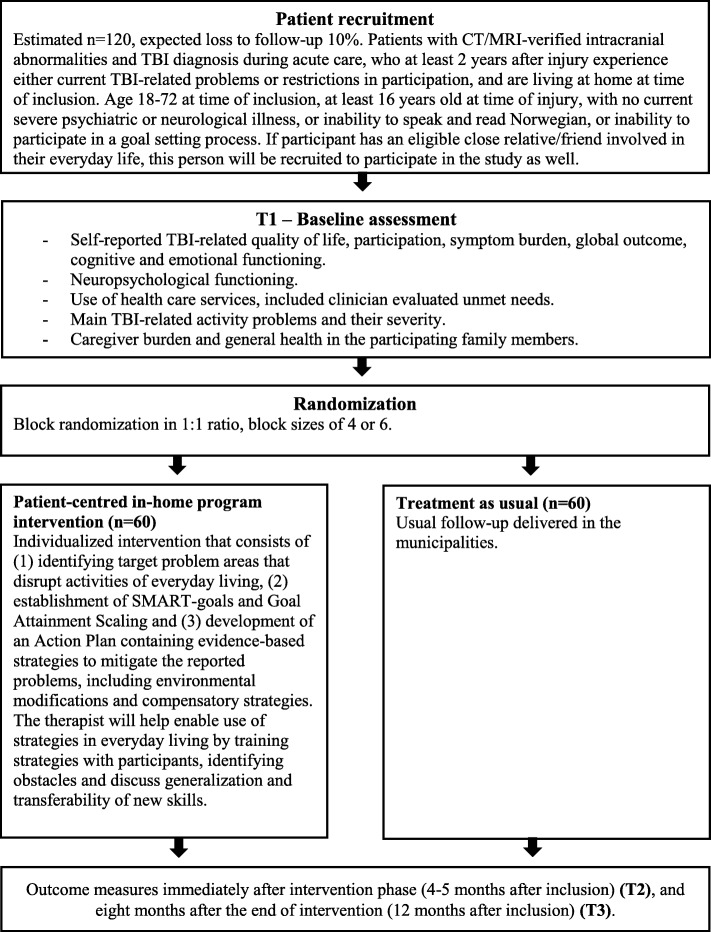
Study flowchart

A study flowchart is provided in Fig. 3.

### Sample size {14}

Sample size calculations were conducted using G*Power [71]. The sample size calculation was based on a power of 0.8 and a *p* value of 0.025 as there are two primary outcomes. Two-sided *t*-tests were used as the basis for the analysis, and a meaningful group difference of 12% for QOLIBRI (pooled SD 20%), and a difference of 1.8 for the Part-O (pooled SD 3), were assumed. With this, 54 patients would be required in each group. With an assumed attrition rate of 10% at T3, 60 participants will be included in each intervention arm.

### Recruitment {15}

Potentially eligible participants will be invited by letter and screened by phone for inclusion and exclusion criteria. Eligibility will be confirmed at baseline assessment before participants are randomized. Potential participants will be recruited from previous research studies conducted at OUH and, if necessary, from the outpatient TBI department at OUH and Sunnaas Rehabilitation Hospital to reach the target sample size.

### Assignment of interventions: allocation

#### Sequence generation {16a}

Participants will be randomly allocated in a 1:1 ratio to either group. A web-based block randomization will be generated by an independent statistician prior to trial start-up to ensure randomization and complete allocation concealment. Variable block size (generated by Stata version 15) will be applied.

#### Concealment mechanism {16b}

The allocation sequence will be stored in a database that can only be accessed by the study principal investigator (PI). Neither the therapists assigning participants to randomization nor the outcome assessors have access to this data base. The PI can only access the numbers sequentially.

### Implementation {16c}

Eligible patients will be identified by the study PI (author CR) from previous studies and the outpatient clinic at OUH. After an initial gross screening by the study PI, further recruitment is performed by the four therapists delivering the intervention. The therapist will assign a randomization number that is different from the study ID number. The randomization number will be sent by web to the study PI who will access the randomization list generated by the statistician to provide information about the allocation and report that to the therapist.

### Assignment of interventions: Blinding

#### Who will be blinded {17a}

Blinding of the participants and therapists is not possible; however, the outcome assessments at T2 and T3 will be conducted by independent assessors blinded to participants’ group assignment. Researcher blinding during statistical analyses will be achieved by reassigning participant ID numbers. To further ensure blinding, an independent researcher will run the main analyses regarding between-group effects.

#### Procedure for unblinding if needed {17b}

There is no need for unblinding procedures in this trial.

### Data collection and management

#### Plans for assessment and collection of outcomes {18a}

Outcome assessors will be trained in the administration of all outcome measures. The estimated time for completion of the data collection is 3–5 h for T1 and 1–2 h for T2 and T3. Most questionnaires and semi-structured interviews used have been translated into Norwegian and validated in previous studies, with a few exceptions. The NPCS is currently being validated in Norwegian conditions. The acceptability scale and Quality of Relationships scale from Winter et al. were translated into Norwegian by our research group and have not yet been validated. Likewise, the Veteran’s In-home Programme Manual developed by Winter et al. was translated into Norwegian and adjusted to the Norwegian setting. The translated manual was evaluated in a feasibility study in which all sessions where conducted by two of the therapists together, ensuring adherence to the manual and reliability as well as identifying necessary adjustments to the Norwegian version before recruitment for the RCT.

#### Plans to promote participant retention and complete follow-up {18b}

One specific researcher has been assigned administrative responsibility for follow-up of all participants to ensure adherence to planned timing of follow-ups (T1, T2, and T3) in both the treatment and control group to ensure call-backs. Any deviation from the standard timing of outcome assessments due to practical or other reasons will be discussed in the study group.

### Data management {19}

All data material will be recorded with a participant ID and will be unidentifiable, and only the researchers working in the project group will have access to lists that link participant numbers with names. De-identified data will be electronically stored on the research server at OUH and will be deleted 5 years after the project has ended. The final dataset will be available to researchers actively contributing to statistical analyses and publications. Data entry will be controlled by initial exploratory analyses, including range checks and double data entry, in order to promote data quality.

### Confidentiality {27}

Information about participants will be handled by health-care professionals adhering to Norwegian law on confidentiality. Information that could contribute to breach of confidentiality will not be published without the express consent of the individuals in question. Data are stored in accordance with Norwegian Data Protection Law.

### Plans for collection, laboratory evaluation, and storage of biological specimens for genetic or molecular analysis in this trial or future use {33}

Not applicable.

### Statistical methods

#### Statistical methods for primary and secondary outcomes {20a}

Descriptive statistics will be used to depict demographics, injury characteristics, and service delivery at baseline as well as acceptability in the intervention group.

The effect of the intervention will be assessed by linear mixed-effect models fitting the primary outcome variables to account for repeated measurements by patients. Time and time-by-treatment interaction will be used as fixed effects in these models. The linear mixed model will give estimated mean values with 97.5% confidence intervals for all time points (T1, T2, and T3) for each group. Estimates of mean between group changes from T1 to T2 and T2 to T3 will also be provided. The analysis of primary interest in establishing treatment efficacy is a time × group interaction in the direction of the intervention group improving above the levels of the control group at T3. Due to two primary outcomes, a significance level of *p* < 0.025 will be applied.

#### Methods for additional analyses (e.g., subgroup analyses) {20b}

Individual and treatment-related predictors for goal attainment will be assessed by multivariable regression analysis in the intervention group. Intention-to-treat analyses will be performed in all analyses adjusted for sociodemographic and service-content variables from the ICSO-R.

### Process evaluation analysis

The participation rate, numbers of consultations, the direct and indirect time related to each consultation, the kinds of problems presented, completion of intervention according to protocol, and any reasons for non-compliance will be assessed. Ten percent of intervention sessions will be overseen by a senior researcher aiming to evaluate treatment fidelity. The participants in the intervention group will rate their degree of belief that the rehabilitation program will help on a scale from 1 to 10 (worst to best) during sessions 1 and 3. After completion of the intervention, the participants and family members will be asked to evaluate the intervention as well as their satisfaction (acceptability).

### Health economic analysis

To determine the cost-effectiveness if the intervention proves to be effective (i.e., at least a moderate effect size on one of the primary outcomes), a statistical analysis of costs will be performed. The total costs will be calculated by adding up direct health-care costs, direct non-health costs, and indirect costs. As the distribution of costs can be skewed, differences in costs between groups will be calculated by means of bootstrapping. A cost-utility analysis will relate the difference between the intervention and control group to changes in utility. This will result in costs per quality-adjusted life years (QALY). QALYs can be derived from the EQ-5D data. Standard discounting will be performed for both costs and outcomes together with sensitivity and uncertainty analyses to gain insight into the generalizability of the economic evaluation.

#### Interim analyses {21b}

No interim analyses will be conducted.

#### Methods in analysis to handle protocol non-adherence and any statistical methods to handle missing data {20c}

Missing data will be handled by multiple imputations for all analyses except the mixed-model analyses, in which missing data will be handled by the analysis using the maximal likelihood approach under the assumption of missing at random.

### Oversight and monitoring

#### Composition of the coordinating centre and trial steering committee {5d}

The translation and adaptation of the intervention program, as well as monitoring of the research process, were performed in close cooperation with the user organization Norwegian Association of People with Injuries, LTN (https://www.personskadeforbundet.no). The Data Protection Office at OUH has reviewed and accepted the trial and will be consulted for any ethical considerations.

#### Composition of the data monitoring committee and its role and reporting structure {21a}

Because of the small size of the study and the timing of the intervention and follow-ups (4 months’ intervention, assessment at 4–5 months and 12 months), we are documenting each intervention and follow-up by date and time to ensure adherence to protocol. Based on this, an external committee was deemed unnecessary.

### Adverse event reporting and harms {22}

Any adverse events will be registered and reported in future publications.

### Frequency and plans for auditing trial conduct {23}

Not applicable.

### Plans for communicating important protocol amendments to relevant parties (e.g., trial participants, ethics committees) {25}

Important protocol modifications will be reported to the Data Protection Office at OUH and amendments will be made to the trial registry (Clinicaltrials.gov).

### Dissemination plans {31a}

Trial reports and other dissemination documents will be written according to the Consolidated Standards of Reporting Trials (CONSORT) statement to facilitate transparency and critical appraisal of the trial [72]. Authorship criteria will adhere to the International Committee of Medical Journal Editors (ICMJE) recommendations [73]. Publications are planned for journals in the fields of neurology, neuropsychology, and rehabilitation. Results will further be disseminated at relevant conferences, national and international meetings, and expert forums. The results will be shared with the user organization and its members as well as policy makers as part of the renewal of rehabilitation services.

## Discussion

This project is innovative in its focus on rehabilitation goals with subjective and long-term relevance to each patient and in the establishment of close collaboration between different levels of health care. The RCT design will enable the establishment of the efficacy of the intervention and, if effective, include a cost-effectiveness analysis. In addition to replicating the effectiveness of the program found by a previous study within a universal health-care system, it will provide knowledge of the suitability of the intervention in civilians living with more severe TBIs, as well as provide information about the effectiveness of the intervention 8 months following treatment. To our knowledge, this is one of the first studies to use a manualized and individualized approach to rehabilitation intervention in the chronic phase of TBI with standardized outcome measures. Hence, the study might potentially have important implications on treatment options and delivery in the chronic phase of TBI that may inform policy and treatment planning [32]. The in-home rehabilitation approach is individually tailored and not only applicable to a TBI population; thus, the findings of this study will bear relevance to other conditions involving chronic neurological deficits and have innovation potential in establishing new modes of collaboration and knowledge transition between specialized acute and post-acute neurosurgical and rehabilitation facilities and rehabilitation services in the municipalities. Users will be involved in all phases of the project, which is in line with recommendations to include users’ perspectives in the development of treatment strategies [74]. The study will also contribute to increased research collaboration among universities, colleges, and primary-care services in the municipalities.

### Limitations

The protocol has several limitations. The individualized nature of the intervention will make it challenging to compare across participants. As previously discussed, however, individualizing the treatment based on the participant’s own goals and competency is a major strength, as it enhances motivation and ensures delivery of relevant treatment. Using the combination of target outcomes, SMART goals, and GAS further enables statistical comparisons across individualized outcomes. Blinding of therapists and participants will not be possible in this study, but outcome assessors and researchers conducting the statistical analyses will remain blinded to group allocation. A further limitation is that all main outcome measures are self-report measurements, which may pose a problem in cases of reduced self-awareness (an issue in all TBI research). However, GAS will be included as a secondary outcome measure in the intervention group, and inclusion of family members when possible will ensure comparable data to assess self-awareness. In addition, the follow-up period of 12 months includes a risk of drop-out. The therapists will be flexible with the timing of interventions and assessments to prevent participants from withdrawing from the study. In the Winter et al. study, dropouts were mainly seen in the participating family members, and a more flexible approach to family member involvement has therefore been adopted in the current study. Further, dropouts will be evaluated as part of the process evaluation.

## Trial status

Protocol version 3.0. Recruitment for the RCT began in June 2018 and will continue until target sample size has been reached, estimated by the end of 2020.

## Data Availability

Not applicable.

## Abbreviations

BRIEF-A: Behavior Rating Inventory of Executive Function Adult Version
CONSORT: Consolidated Standards of Reporting Trials
CT: Computed tomography
CVLT: California Verbal Learning Test
D-KEFS: Delis-Kaplan Executive Function Systems
GAD-7: Generalized Anxiety Disorder seven-item scale
GAS: Goal Attainment Scaling
GOS-E: Glasgow Outcome Scale-Extended
ICMJE: International Committee of Medical Journal Editors
ICSO-R: International Classification System for Service Organization in Health-related Rehabilitation
MRI: Magnetic resonance imaging
NPCS: Needs and Provision Complexity Scale
OUH: Oslo University Hospital
PART-O: Participation Assessment with Recombined Tools-Objective
PHQ-9: Patient Health Questionnaire-9
QALY: Quality-adjusted life years
RCT: Randomized controlled trial
REK: Regional Ethics Committee
RPQ: Rivermead Post-concussion Questionnaire
SPIRIT: Standard Protocol Items: Recommendations for Interventional Trials
TAU: Treatment as usual
TBI: Traumatic brain injury
WAIS-IV: Weschler Adult Intelligence Scale-fourth edition
